# Dosing of ritonavir-boosted darunavir for treatment of HIV in pregnancy

**DOI:** 10.1097/QAD.0000000000004461

**Published:** 2026-02-11

**Authors:** Rebecca Sconza, Graham P. Taylor, Luminita Ene, Christian Kahlert, Lena van der Wekken-Pas, Françoise Renaud, Claire Thorne, Claire L. Townsend

**Affiliations:** a UCL Great Ormond Street Institute of Child Health, University College London; b Section of Virology, Department of Infectious Disease, Faculty of Medicine, Imperial College London, London, UK; c “Dr Victor Babes” Hospital of Infectious and Tropical Diseases, Bucharest, Romania; d Children's Hospital of Eastern Switzerland, St Gallen, Switzerland; e Department of Pharmacy, Pharmacology and Toxicology, Radboud Institute for Medical Innovations (RIMI), Radboud University Medical Center, Nijmegen, The Netherlands; f Department of HIV, TB, Hepatitis and STI Programmes, World Health Organization, Geneva, Switzerland.

**Keywords:** antiretroviral agent, darunavir, HIV protease inhibitor, infectious disease transmission, pregnancy, treatment outcome, vertical

## Abstract

**Objective:**

To assess the effectiveness of once- and twice-daily ritonavir-boosted darunavir (DRV/r)-containing regimens for treating HIV in pregnancy to inform the 2025 World Health Organization antiretroviral treatment guidelines update.

**Design:**

Analysis of pooled data from two observational study networks with sites in Romania, Switzerland, and the United Kingdom.

**Methods:**

Pregnancies resulting in a live birth or stillbirth in women receiving 800/100 mg DRV/r once-daily or 600/100 mg twice-daily during pregnancy were included. The primary outcome was viral suppression (<50 copies/ml) near delivery (from 28 days prior to 7 days after delivery).

**Results:**

Among 162 women on once-daily DRV/r, 95% were virally suppressed near delivery, with no difference between those on DRV/r from conception (95%, 113/119) and those who started on or switched to DRV/r during pregnancy (95%, 41/43). Among 27 women on twice-daily DRV/r, 78% were virally suppressed near delivery. Most women remained on the same DRV/r regimen until delivery, and there were no vertical transmissions. Darunavir drug concentrations for the limited number of pregnancies with data available fell within the expected ranges.

**Conclusions:**

This analysis provides some reassurance that once-daily DRV/r can be used successfully in pregnancy. However, given the possibility of reduced drug levels in pregnancy with once-daily dosing, viral load monitoring during pregnancy remains essential. Surveillance of pregnancies in women receiving once-daily DRV/r is needed to further support the use of this dosing during pregnancy.

## Introduction

Darunavir (DRV) is a highly effective and well tolerated protease inhibitor (PI) with a high genetic barrier to resistance used for treating antiretroviral therapy (ART)-naïve and -experienced people living with HIV-1 [1]. Prior to 2025, ritonavir-boosted darunavir (DRV/r)-based ART was recommended by the World Health Organization (WHO) as an alternative option for second-line treatment, with two other ritonavir-boosted PIs, atazanavir (ATV/r) and lopinavir (LPV/r), considered preferred options [2]. DRV/r has a superior tolerability and efficacy profile compared with ATV/r and LPV/r [3–5], but lack of affordable, generic options and of a fixed-dose co-formulation of DRV/r limited its use in low- and middle-income settings.

The development of a generic DRV/r co-formulation in 2022 [6], alongside evidence of toxicities related to other PIs, made DRV/r a favourable option to replace ATV/r and LPV/r as preferred PI for subsequent ART regimens in 2025 updated WHO recommendations [7]. However, a key component of the WHO ART drug optimization approach is harmonizing regimens across populations, including children, adolescents, and pregnant women [8]. DRV/r-based ART is considered safe and effective in pregnancy [9–12], with some comparative data suggesting superior viral efficacy compared with ATV/r when DRV/r is given twice daily (600/100 mg b.i.d.) [11], but understanding of its safety and efficacy in pregnancy at the standard once-daily dose (800/100 mg q.d.) has been limited.

In nonpregnant adults, once-daily DRV/r is recommended in the absence of resistance mutations while twice-daily administration is recommended for those with history of PI resistance and/or adherence issues [13–15]. In pregnancy, PIs including DRV/r have been shown to have reduced drug concentrations in the third trimester compared with postpartum and nonpregnant adults, although the clinical relevance of this is uncertain. Data from small pharmacokinetics studies showed that the once-daily dose resulted in a greater decrease in both total and unbound DRV in the second and third trimester than a twice-daily dose [16–21]. For this reason, most guidelines recommend twice-daily dosing in pregnancy for women starting DRV/r during pregnancy, with once-daily dosing considered appropriate in some settings for women who conceive on DRV/r and are virally suppressed [13–15]. Cobicistat-boosted DRV (DRV/c) is not recommended in pregnancy due to significant decreases in DRV concentrations throughout pregnancy induced by cobicistat and consequent reduced viral efficacy [22,23].

To date, no studies have compared viral efficacy of DRV/r once- vs. twice-daily in pregnancy, and studies reporting outcomes among those receiving once-daily DRV/r have been limited by small sample sizes [10,12]. The aim of this analysis was to describe DRV/r dosing regimens in pregnant women and assess the effectiveness of once- and twice-daily DRV/r-based ART for treating HIV in pregnancy and preventing vertical transmission using real-world European data in order to inform 2025 WHO guidelines updates.

## Methods

We conducted a pooled analysis of pseudonymized individual-patient data on pregnancies in women with HIV-1 from two retrospective observational study networks: the Epidemiology of Pregnancy and Paediatric Infections International Cohort Collaboration (EPPICC) and the London HIV Perinatal Research Group (LHPRG). Pregnancies with any exposure to DRV/r resulting in a live birth or stillbirth at ≥22 completed weeks’ gestation up to December 2024 were eligible for inclusion. Participating EPPICC cohorts included the Swiss Mother and Child HIV Cohort Study (MoCHiV), a multicentre study collecting pregnancy data for all women living with HIV enrolled in the Swiss HIV Cohort Study [24], and the Victor Babes Hospital Cohort, a single-centre cohort comprising all pregnant women living with HIV receiving care at the “Dr Victor Babes” Hospital of Infectious and Tropical Diseases in Bucharest, Romania. The LHPRG contributed data on all eligible pregnancies across 11 National Health Service (NHS) hospitals in London, UK.

All data were originally collected per routine clinical care/study procedures and later submitted to the EPPICC Pregnancy coordinating centre at the UCL Great Ormond Street Institute of Child Health for data pooling and analysis using a standard operating procedure based on a modified HIV Cohorts Data Exchange Protocol (https://hicdep.org/). Pregnancies missing data on DRV/r dose or timing were excluded.

Viral effectiveness analyses were restricted to pregnancies in which standard DRV/r doses (i.e., 800/100 mg once daily or 600/100 mg twice daily) were given, without the use of other co-administered anchor agents [PIs, integrase strand transfer inhibitors (INSTIs), nonnucleoside reverse transcriptase inhibitors (NNRTIs), or entry inhibitors]. Our main study outcome was viral suppression near delivery, defined as last viral load in pregnancy <50 copies/ml, measured between 28 days prior to and 7 days after delivery. We compared viral suppression near delivery by DRV/r dose received, stratified by key pregnancy and treatment characteristics. Four DRV/r treatment groups were defined:

(1) DRV at conception (including pregnancies conceived on DRV/c and subsequently switched to DRV/r)
(2) Other ART at conception, switched to DRV/r during pregnancy
(3) No ART at conception, initiated DRV/r during pregnancy
(4) No ART at conception, initiated on other ART during pregnancy and switched to DRV/r

Pregnancies exposed to both once- and twice-daily doses in the same pregnancy were described separately.

DRV plasma concentrations during pregnancy were available from some London hospitals where antenatal therapeutic drug monitoring of PIs was performed routinely. Darunavir and ritonavir concentrations were quantified using a ULPC–MS/MS assay (lower limit of quantification: 39 ng/ml). Concentrations measured within 8 and 18 h after drug intake in women receiving twice vs. once daily DRV, respectively, were considered eligible for analysis, as these measurements best reflect trough concentrations. For nondetectable concentrations, 50% of the lowest level of quantification (19 ng/ml) of the assay was taken to impute these values.

Other outcomes included preterm delivery (<37 weeks), stillbirth (intrauterine death ≥22 weeks), and vertical HIV transmission. Vertical transmission was described among singleton infants with confirmed HIV infection status at the time of data pooling, as reported by cohorts.

Measures of frequency were summarized for categorical variables, and continuous variables with nonparametric distribution were summarized using median and interquartile range (IQR). Associations between categorical variables were assessed using chi-squared or Fisher's exact tests, and differences in medians using the Mann–Whitney test. A sensitivity analysis was conducted to explore the effect of excluding pregnancies where women switched from DRV/c to DRV/r.

EPPICC cohorts obtained ethics approval from local committees for participation in EPPICC Pregnancy data mergers and projects. EPPICC Pregnancy has research ethics approval from the University College London Research Ethics Committee (reference 3715.007). Data obtained from LHPRG were anonymized prior to analysis and used in accordance with National Research Ethics Service guidance.

Data management and analyses were conducted in Stata 18.5 MP (College Station, Texas, USA).

## Results

From a total of 336 DRV/r-exposed pregnancies with birth outcomes in 2008–2024, 244 were in women who received standard DRV/r doses without additional anchor agents; 198 (81%) of these received DRV/r 800/100 mg once-daily and 46 (19%) DRV/r 600/100 twice-daily. Among the remainder, there were 78 pregnancies where DRV/r was only ever given in a nonstandard dose or co-administered with another anchor agent (usually an INSTI) and 14 with exposure to both once- and twice-daily standard DRV/r doses (without co-administered anchor drugs).

### Pregnancies exposed to once- or twice-daily darunavir/ritonavir dosing

#### Pregnancy and treatment characteristics

Characteristics of the 244 pregnancies included in the effectiveness analyses are presented in Table 1. Two-thirds of pregnancies (64%, 157/244) were reported from London. Eleven percent (26/244) of pregnancies were in women diagnosed during the current pregnancy, and 21% (52/244) started ART during pregnancy (Table 1). For most (88%), DRV/r was first given with a nucleoside backbone of tenofovir disoproxil fumarate plus emtricitabine (TDF+FTC) (69%, 168/244) or abacavir plus lamivudine (ABC+3TC) (19%, 47/244).

**Table 1:** Pregnancy characteristics by standard DRV/r dose received, *N* = 244.

	DRV/r 800/100 mg once daily, *N* = 198	DRV/r 600/100 mg twice daily, *N* = 46	Total, *N* = 244	*P*-value
Age at delivery, years, median (IQR)	35 (31–38)	29 (27–34)	34 (29–38)	<0.001
Cohort country, *n* (%)				
Romania	0 (0.0)	18 (39.1)	18 (7.4)	
Switzerland	62 (31.3)	7 (15.2)	69 (28.3)	
United Kingdom	136 (68.7)	21 (45.7)	157 (64.3)	<0.001
Timing of HIV diagnosis, *n* (%)				
Before this pregnancy	184 (92.9)	34 (73.9)	218 (89.3)	
During this pregnancy	14 (7.1)	12 (26.1)	26 (10.7)	<0.001
ART timing^a^, *n* (%)				
Conceived on ART	169 (85.4)	23 (50.0)	192 (78.7)	
Started ART in 1st/2nd trimester	27 (13.6)	16 (34.8)	43 (17.6)	
Started ART in 3rd trimester	2 (1.0)	7 (15.2)	9 (3.7)	<0.001
DRV group, *n* (%)				
DRV at conception	143 (72.2)	18 (39.1)	161 (66.0)	
Other ART at conception, switched to DRV/r	26 (13.1)	5 (10.9)	31 (12.7)	
Started ART in pregnancy (DRV/r)	25 (12.6)	20 (43.5)	45 (18.4)	
Started ART in pregnancy (other ART, switched to DRV/r)	4 (2.0)	3 (6.5)	7 (2.9)	<0.001
NRTI backbone, first DRV/r regimen, *n* (%)				
TDF+FTC	142 (71.7)	26 (56.5)	168 (68.9)	
TDF+3TC	0 (0.0)	6 (13.0)	6 (2.5)	
ABC+3TC	45 (22.7)	2 (4.3)	47 (19.3)	
Other	11 (5.6)	12 (26.1)	23 (9.4)	<0.001
Subsequent regimen change from DRV/r, *n* (%)				
No	182 (91.9)	40 (87.0)	222 (91.0)	
Yes	16 (8.1)	6 (13.0)	22 (9.0)	0.267
First CD4^+^ cell count in pregnancy, cells/mm^3^, median (IQR) (*n* = 238)	527 (386–707)	448 (317–588)	511 (370–686)	0.033
First CD4^+^ cell count in pregnancy, *n* (%) (*n* = 238)				
<350 cells/mm^3^	39 (20.3)	17 (37.0)	56 (23.5)	
≥350 cells/mm^3^	153 (79.7)	29 (63.0)	182 (76.5)	0.017
Prior virological failure on PIs, *n* (%) (*n* = 212)				
No	155 (89.1)	33 (86.8)	188 (88.7)	
Yes	19 (10.9)	5 (13.2)	24 (11.3)	0.777
PI resistance, *n* (%) (*n* = 147)				
No	109 (88.6)	17 (70.8)	126 (85.7)	
Yes	14 (11.4)	7 (29.2)	21 (14.3)	0.049
HBV surface antigen positive, *n* (%) (*n* = 236)				
No	182 (95.3)	38 (84.4)	220 (93.2)	
Yes	9 (4.7)	7 (15.6)	16 (6.8)	0.017
Pregnancy outcome, *n* (%)				
Livebirth	197 (99.5)	46 (100.0)	243 (99.6)	
Stillbirth	1 (0.5)	0 (0.0)	1 (0.4)	1.000
GA at delivery, completed weeks, median (IQR)	39 (38–40)	38 (37–39)	38 (38–40)	0.007
Preterm delivery (<37 weeks), *n* (%)				
No	168 (84.8)	41 (89.1)	209 (85.7)	
Yes	30 (15.2)	5 (10.9)	35 (14.3)	0.460
Delivery VL, *n* (%) (*n* = 189)				
<50 copies/ml	154 (95.1)	21 (77.8)	175 (92.6)	
≥50 copies/ml	8 (4.9)	6 (22.2)	14 (7.4)	0.007

a Overall ART summary, not specific to the DRV/r-based regimen received.

3TC, lamivudine; ABC, abacavir; ART, antiretroviral therapy; DRV, darunavir; DRV/r, ritonavir-boosted darunavir; FTC, emtricitabine; GA, gestational age; HBV, hepatitis B virus; IQR, interquartile range; NRTI, nucleoside reverse transcriptase inhibitor; PI, protease inhibitor; TDF, tenofovir disoproxil fumarate; VL, viral load.

Note: This table excludes 14 pregnancies exposed to both 800/100 mg once-daily and 600/100 mg twice-daily DRV/r.

Of the 161 (66%) pregnancies in women on DRV at conception, boosting was initially with cobicistat (DRV/c) in 26; this was changed to DRV/r for all women, at a median of 12 completed weeks’ gestation (IQR: 8–16; range: 5–29). One woman conceived on DRV/r, switched to DRV/c at 7 weeks, and switched back to DRV/r at 10 weeks. Among the 31 (13%) women who were on non-DRV-based regimens at conception and switched to DRV/r, 11 switched from other PI-based regimens, 10 from NNRTI-based regimens, seven from INSTI-based regimens (including 6 on dolutegravir), and three from other regimens; switch to DRV/r occurred at a median of 10 completed weeks’ gestation (IQR: 5–19, range: 1–35). Where women started ART in pregnancy on non-DRV-based regimens and switched to DRV/r (3%, 7/244), five switched from other PI-based regimens and two from INSTI-based regimens; median gestational age at DRV/r start was 23 completed weeks’ gestation (IQR: 16–32; range: 7–37).

There were no pregnancies in women on once-daily DRV/r reported from the Romanian cohort. Compared with those on once-daily DRV/r, women on twice-daily DRV/r were significantly younger, more likely to be diagnosed in the current pregnancy (26% vs. 7%, *P* < 0.001), and more likely to have started ART during pregnancy as opposed to before (50% vs. 15%, *P* < 0.001) (Table 1). They were also more likely than women on once-daily DRV/r to have a first CD4^+^ cell count in pregnancy < 350 cells/mm^3^ (37% vs. 20%, *P* = 0.017), to have PI resistance (29% vs. 11%, *P* = 0.049), and to be hepatitis B virus (HBV) surface antigen positive (16% vs. 5%, *P* = 0.017) (Table 1).

Of 21 women with known history of PI resistance, 19 had no DRV resistance mutations, one had increased DRV susceptibility (on once-daily DRV/r), and one had low-level DRV resistance (on twice-daily DRV/r). In 15 of the 21 women, HIV was susceptible to DRV, lopinavir, and atazanavir, with mutations only impacting older PIs.

#### Viral suppression near delivery

Among the 78% (189/244) of pregnancies with viral load data available, viral suppression (<50 copies/ml) near delivery occurred in 95% (154/162) of those on once-daily DRV/r and 78% (21/27) of those on twice-daily DRV/r (*P* = 0.001) (Table 1). In univariable analysis, other factors associated with viral suppression near delivery (*P* ≤ 0.1) included conceiving on ART (95%, 142/150, vs. 85%, 33/39, for starting during pregnancy), having a first CD4^+^ cell count in pregnancy ≥350 cells/mm^3^ (94%, 132/140, vs. 86%, 38/44, for CD4^+^ cell count <350 cells/mm^3^), and being from the Swiss cohort (98%, 49/50, vs. 91%, 124/136, for the London cohort and 67%, 2/3, for the Romanian cohort) (Table S1, Supplemental Digital Content). The small number of women on twice-daily DRV/r (*n* = 27) precluded multivariable analyses.

**Fig. 1:**
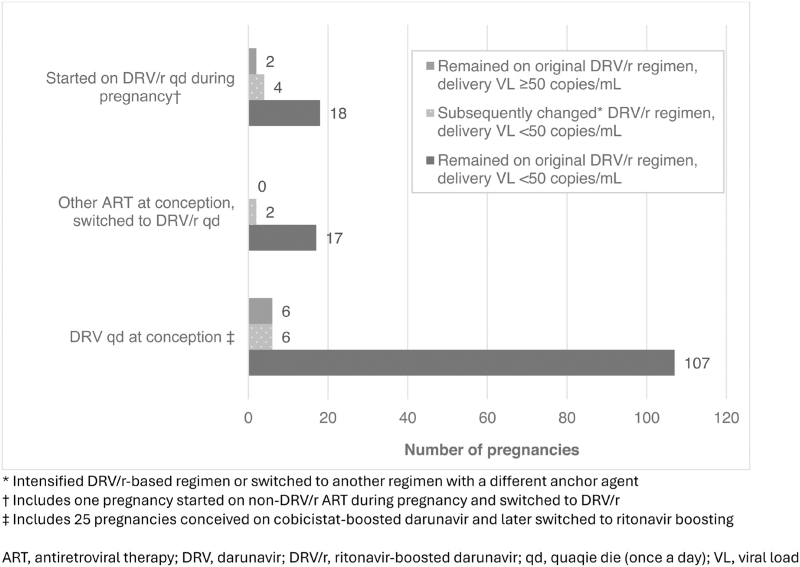
Regimen change status in pregnancy and delivery viral load by treatment group, darunavir/ritonavir 800/100 mg once daily.

Among women on once-daily DRV/r, there were no differences in the proportion with viral suppression near delivery across the treatment groups (DRV/r from conception, 95%; started during pregnancy, 91%; switched to DRV/r during pregnancy, 100%) (Table 2). Among all 43 pregnancies where women started on or switched to once-daily DRV/r in pregnancy, median timing of initiation of DRV/r was 14 completed weeks’ gestation (IQR, 7–20; range, 1–34); one woman started DRV/r in the third trimester at 34 weeks as part of a switch from another regimen with undetectable viral load; overall 95% (41/43) were virally suppressed near delivery. Among women on once-daily DRV/r with viral load available, 93% (150/162) remained on the same regimen, while 7% (12/162) either switched to another regimen (*n* = 5) or added an additional anchor agent (raltegravir or dolutegravir, *n* = 7) before delivery (Fig. 1).

**Table 2:** Viral suppression near delivery by DRV/r dose and maternal and pregnancy characteristics, *N* = 189.

	DRV/r 800/100 mg once daily, *N* = 162		DRV/r 600/100 mg twice daily, *N* = 27	
		
	Total	Delivery VL <50 copies/ml, *n* (%)	Total	Delivery VL <50 copies/ml, *n* (%)
All pregnancies	162	154 (95.1)	27	21 (77.8)
DRV group				
DRV at conception	119	113 (95.0)	8	8 (100.0)
Other ART at conception, switched to DRV/r	19	19 (100.0)	4	2 (50.0)
Started ART in pregnancy (DRV/r)	23	21 (91.3)	13	9 (69.2)
Started ART in pregnancy (other ART, switched to DRV/r)	1	1 (100.0)	2	2 (100.0)
Cohort country				
Romania	0		3	2 (66.7)
Switzerland	45	45 (100.0)	5	4 (80.0)
United Kingdom	117	109 (93.2)	19	15 (78.9)
First CD4 count in pregnancy (*n* = 184)				
<350 cells/mm^3^	33	31 (93.9)	11	7 (63.6)
≥350 cells/mm^3^	124	118 (95.2)	16	14 (87.5)
ART timing				
Conceived on ART	138	132 (95.7)	12	10 (83.3)
Started ART in 1st/2nd trimester	22	21 (95.5)	10	6 (60.0)
Started ART in 3rd trimester	2	1 (50.0)	5	5 (100.0)
Prior virological failure on PIs (*n* = 170)				
No	129	123 (95.3)	23	19 (82.6)
Yes	15	15 (100.0)	3	1 (33.3)
PI resistance (*n* = 126)				
No	97	91 (93.8)	14	12 (85.7)
Yes	10	10 (100.0)	5	3 (60.0)
Preterm delivery (<37 weeks)				
No	138	134 (97.1)	24	18 (75.0)
Yes	24	20 (83.3)	3	3 (100.0)

ART, antiretroviral therapy; DRV, darunavir; DRV/r, ritonavir-boosted darunavir; PI, protease inhibitor; VL, viral load.

Note: 12/162 (7%) on once-daily and 6/27 (22%) on twice-daily switched off or intensified the DRV/r-based regimen prior to delivery.

The proportion with viral suppression near delivery was not significantly different in those with and without prior virological failure on PIs (100% and 95%, respectively, *P* = 0.39), or in those with and without reported PI resistance (100% and 94%, respectively, *P* = 0.42; data only available for 66% of pregnancies) (Table 2). There was, however, a significant association between preterm delivery and detectable delivery viral load: 83% of those delivering preterm were virally suppressed near delivery vs. 97% of term deliveries (*P* = 0.02). A third (33%, 8/24) of those delivering preterm had initiated ART during the current pregnancy, compared with 12% (16/138) of those delivering at term (*P* = 0.006).

Among women on DRV/c at conception who switched to DRV/r during pregnancy, 92% (23/25) were virally suppressed near delivery. In a sensitivity analysis excluding these pregnancies, associations between maternal and pregnancy characteristics (including treatment group) and virological outcome were not substantially altered.

The eight detectable viral loads near delivery among women on once-daily DRV/r ranged from 64 to 1369 copies/ml (4/8 were <200 copies/ml; 7/8 <1000 copies/ml). Four women had several undetectable viral loads reported during pregnancy, followed by a single low-level detectable viral load at or near delivery; one of these women was on DRV/c from conception to 29 weeks, then switched to DRV/r and delivered at 39 weeks. Two women had low-level viraemia throughout pregnancy: one delivered at 29 weeks, while the other had several undetectable viral loads during pregnancy, including at 38 weeks, and delivered at term with a detectable viral load. The remaining two women (one diagnosed before, one during pregnancy) started treatment with DRV/r (at 20 and 31 weeks, respectively), had low or falling viral loads, and delivered preterm (at 32 and 34 weeks, respectively). Overall, four of the eight women with detectable viral load at delivery delivered preterm, at 29–36 weeks. No PI resistance or previous virological failure on PIs was reported among the six women with information available.

Among the pregnancies in women on twice-daily DRV/r, 29% (6/21) of those virally suppressed near delivery subsequently switched (*n* = 2) or intensified their regimen with an INSTI (*n* = 4) before delivery.

#### Vertical transmission

There were no vertical transmissions among the 243 pregnancies resulting in live births in women on DRV/r once- or twice-daily regimens in pregnancy (5/249 live born infants had unknown or indeterminate infection status) (95% confidence limit for vertical transmission rate among 231 singleton infants, 1.6%). Of note, there were also no transmissions among the 92 pregnancies excluded from the effectiveness analyses (3/94 live born infants had unknown or indeterminate infection status).

### Pregnancies exposed to both once- and twice-daily darunavir/ritonavir dosing

Among the 14 women exposed to both once- and twice-daily dosing, 10 conceived on once-daily DRV/r, one had conceived on another regimen and switched to once-daily DRV/r in pregnancy, and three had started once-daily DRV/r in pregnancy. Switching occurred at a median of 27 weeks’ gestation (IQR: 24–30; range: 5–37). Reason for switching to twice-daily DRV/r was given as low-level viraemia for two, and a further two had documented low-level viraemia at the time of switch; two of these four women were virally suppressed near delivery. Overall, 10 of 12 women were virally suppressed near delivery (two with missing delivery viral load were suppressed within six weeks of delivery).

### Darunavir/ritonavir drug concentrations during pregnancy

**Fig. 2:**
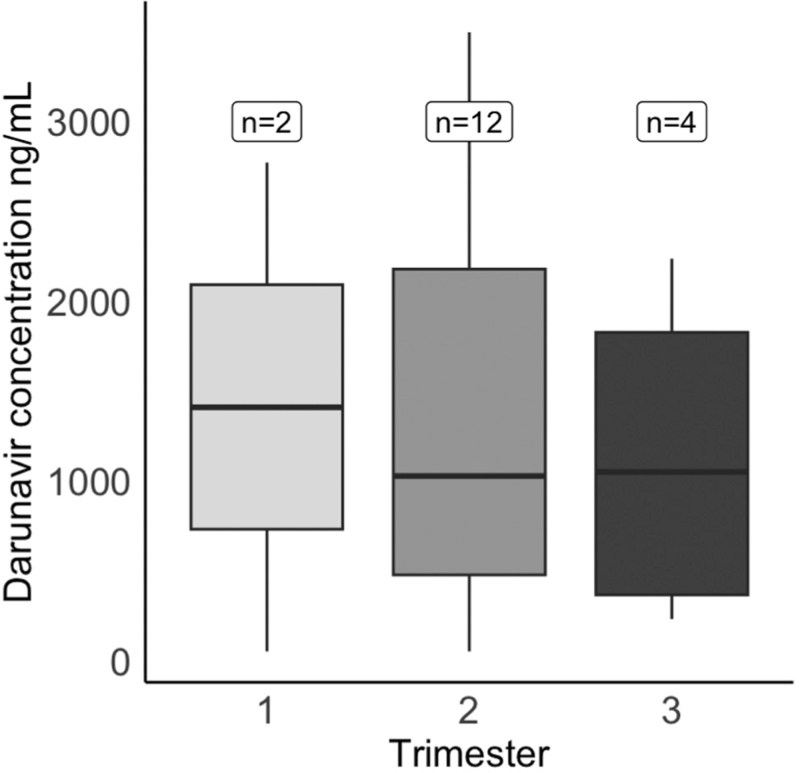
Plasma trough concentrations (mg/l) by trimester of measurement, darunavir 800 mg once daily.

DRV plasma concentrations were available for 18 pregnancies on once-daily DRV/r and four on twice-daily DRV/r (all from the UK). Median DRV trough concentration (*C*_trough_) in those on once-daily dosing was 1028.5 ng/ml (IQR: 469.8–2178.2) for measurements in the second trimester (*n* = 12) and 1051.0 ng/ml (IQR: 367.2–1826.5) for those in the third trimester (*n* = 4) (Fig. 2). Two samples from women who received once daily dosing had undetectable concentrations, whereas all samples from women who received twice daily dosing had detectable concentrations.

## Discussion

Across cohorts in the United Kingdom, Switzerland, and Romania, most pregnant women on standard DRV/r-based regimens received a once-daily dose of 800/100 mg DRV/r. Among 162 women on the once-daily regimen, 95% were virally suppressed (<50 copies/ml) near delivery and there were no vertical transmissions. Although nearly three-quarters of women on once-daily DRV/r had started DRV/r before conception, there was no difference in viral suppression near delivery between those on DRV/r from conception and those who started or switched onto DRV/r during pregnancy. Once conceived or started on once-daily DRV/r, most women (93%) remained on this dose for the duration of pregnancy, with no other switches. Eight women on once-daily DRV/r had detectable viral loads near delivery, although four delivered prematurely, two of whom had started treatment during pregnancy and therefore had limited time for treatment response to occur.

Our findings are in line with two other observational studies of once-daily DRV/r in pregnancy conducted in North America, in which viral suppression rates (<20 or <40 copies/ml) of 83% (19/23) and 94% (32/34) were reported [10,12]. In one small single-centre study, there was no significant difference in the probability of viral suppression by delivery in adjusted analyses between those receiving once-daily DRV/r- and dolutegravir-based regimens [10]. We were not powered in our study to carry out meaningful adjusted analysis to directly compare viral outcomes by DRV/r dose received (due to our small sample of 27/46 pregnancies on the twice-daily dose with viral load data available, with substantial differences in baseline and treatment characteristics from the once-daily group). However, the level of viral suppression we report here among pregnant women on once-daily DRV/r is comparable to that seen among pregnant women on twice-daily DRV/r in a recent PHACS/SMARTT study, where 90% (128/142) achieved viral load <200 copies/ml near delivery (with no statistically significant differences between DRV/r- and dolutegravir-based regimens) [11].

Although viral suppression near delivery among women conceiving on DRV/c and later switching to once-daily DRV/r was similar to other treatment groups, reduced DRV concentrations in pregnancy with cobicistat boosting are well documented [22,23], and switching from cobicistat to ritonavir boosting should occur as soon as possible after pregnancy is confirmed [13]. Most women on DRV/c in this study switched to DRV/r by the fourth month of pregnancy, but approximately a quarter did not switch until well into the second or third trimester. Given the potential for delay to switch to ritonavir boosting in pregnancy, providers should consider the reproductive potential of women living with HIV when prescribing cobicistat-boosted ART and provide appropriate counselling around its safety in pregnancy.

Despite overall successful clinical outcomes with once-daily DRV/r, there were a few instances of low-level viraemia near delivery among women on once-daily DRV/r with previously undetectable viral load. In addition, among women switching from once- to twice-daily DRV/r, four had low-level viraemia at the time of switch, and in two of these, this was given as the reason for switching. Although information on adherence was lacking, we cannot rule out low drug concentrations as the reason for the increase in viral load. Based on our limited data on DRV plasma concentrations during pregnancy (obtained solely from the London cohort), therapeutic DRV levels did not appear to be substantially reduced in the second and third trimesters, although only four data points were available in the third trimester. These findings are in line with those reported in the PANNA study, in which all 24 women on DRV/r in pregnancy had drug concentrations above the therapeutic drug target for wild-type virus (EC_50_), with all but one having levels above the target for resistant virus in the second and third trimesters [18,25]. However, given that drug concentration levels are lower in the latter part of pregnancy, adherence support and routine viral load monitoring during pregnancy remain essential with once-daily dosing. One study reported that although most women had >75% adherence to DRV/r-based ART, those with <75% adherence had multiple detectable viral loads reported during pregnancy [12].

Protease mutations were uncommon in this study and mostly did not impact susceptibility to DRV or the currently available alternatives, atazanavir and lopinavir. This is likely to represent the situation addressed by the new WHO guidelines where people living with HIV have been treated with dolutegravir-based ART and have had little or no exposure to PIs, especially DRV. When DRV/r is prescribed following treatment failure on a PI, viral load monitoring should be monitored closely where possible.

To our knowledge, this is the largest study of pregnancies in women receiving once-daily DRV/r-based regimens, with representation from both Western and Eastern Europe. The two study networks involved are established collaborations with robust data collection protocols, and the collaborative framework provided by EPPICC allowed us to conduct this analysis rapidly, in response to a specific policy question and supporting decision-making for the updated WHO guidelines launched in July 2025 [7].

The unexpectedly small sample size for twice-daily dosing prevented us from directly comparing the effectiveness of the two dosing approaches. Other limitations included a lack of information on treatment adherence and concomitant medications, which may have influenced the choice of regimen or dose or led to regimen changes. Given the little resistance observed, we were also unable to address DRV/r dosing where DRV resistance was known or suspected. Treatment histories of the women in these cohorts were highly variable since DRV/r was frequently used as a third-line regimen in the earlier study years [26]. Unfortunately, there were limited data on use of DRV/r following treatment failure or intolerance to dolutegravir-based regimens. Although these data provide some reassurance, it will be important to monitor treatment responses in women switching from currently recommended antiretrovirals such as dolutegravir to once-daily DRV/r during pregnancy. While data on ART in pregnancy are collected in several prospective surveillance and observational studies, information on dosing is not always routinely collected. Additional efforts may be needed to capture such data in the future.

## Conclusion

This analysis suggests that once-daily DRV/r can be used successfully in pregnancy. However, uncertainty remains around the possibility of low-level viraemia resulting from low drug concentrations in pregnancy, highlighting the importance of viral load monitoring during pregnancy. There is an ongoing need for robust surveillance of once-daily DRV/r use in pregnant women, particularly given the new WHO recommendation for DRV/r as the preferred PI option for subsequent regimens for individuals whose dolutegravir-based regimen is failing [7].

## Acknowledgements

EPPICC author contributors: Karoline *Aebi-Popp* (Department of Infectious Diseases, Bern University Hospital, University of Bern, Bern, Switzerland), Heather *Bailey* (UCL Institute for Global Health, University College London, London, UK), Giorgia *Dalla Valle* (Penta Foundation, Padua, Italy).

LHPRG author contributors: Emily *Cheserem* (North Middlesex University Hospital NHS Trust, London, UK), Anette *Elbech* (Chelsea and Westminster Hospital NHS Foundation Trust, London, UK), Kay *Francis* (North Middlesex University Hospital NHS Trust, London, UK), Rahal *Fernando* (Imperial College Healthcare NHS Trust, London, UK), Dawn *Friday* (London North West University Healthcare NHS Trust, London, UK), Jessica *Gaddie* (Barts Health NHS Trust, London, UK), Eleanor *Hamlyn* (Royal Free London NHS Foundation Trust, London, UK), Joseph *Heskin* (Chelsea and Westminster Hospital NHS Foundation Trust, London, UK), Suki *Leung* (Chelsea and Westminster Hospital NHS Foundation Trust, London, UK), Emily *Mabonga* (Lewisham and Greenwich NHS Trust, London, UK), Sherie *Roedling* (Central and North West London NHS Foundation Trust, London, UK), Shiv *Shah* (London North West University Healthcare NHS Trust, London, UK), Rebecca *Simons* (Guy's and St Thomas’ NHS Foundation Trust, London, UK), Brenton *Wait* (Homerton Healthcare NHS Foundation Trust, London, UK), Yasmin *Walters* (Chelsea and Westminster Hospital NHS Foundation Trust, London, UK), Claire *Williams* (Guy's and St Thomas’ NHS Foundation Trust, London, UK).

C.T. and G.P.T. conceptualized the study. Development of the study data specification and analysis plan was led by R.S., with input from C.T., G.P.T., F.R., and C.L.T. All EPPICC and LHPRG authors were involved in the collection of the data. R.S. carried out all study data management and performed the statistical analyses. L.W. carried out the plasma concentrations analyses. R.S. and C.L.T. drafted the manuscript, with input from G.P.T., F.R., and C.T. All authors contributed to interpretation of study findings and preparation and review of the final manuscript.

We thank Ali Judd and Intira Jeannie Collins (EPPICC co-leads, Medical Research Council Clinical Trials Unit at UCL), Georgina Fernandes (EPPICC Pregnancy doctoral researcher, UCL Great Ormond Street Institute of Child Health), and all those who supported the collection and preparation of EPPICC Pregnancy cohort data: Katharina Kusejko and Katja Benic (Swiss MoCHiV) and Roxana Radoi (Victor Babes Cohort).

We gratefully acknowledge members of the WHO HIV, Hepatitis and STIs Pregnancy and Breastfeeding Therapeutics Working Group for their input on the concept for this study and feedback on preliminary results, particularly Lynne Mofenson, Shahin Lockman, Elaine Abrams, and Angela Colbers.

This work is supported by the National Institute for Health Research (NIHR) Great Ormond Street Hospital Biomedical Research Centre. The views expressed are those of the authors and not necessarily those of the NHS, the NIHR, or the Department of Health.

### Conflicts of interest

Conflicts of interest and source of funding: EPPICC has received funding from ViiV Healthcare and Merck Sharpe & Dohme via Penta Foundation. CT reports funding/grants from NHS England, European Union Horizon Europe, Child Health Research CIO, and Penta Foundation. LW has received grants from ZonMW and Merck and Gilead (paid to institution). There were no other conflicts (RS, GPT, LE, CK, FR, CLT). Some of this work was undertaken at UCL Great Ormond Street Institute of Child Health, which receives a proportion of funding from the UK Department of Health's National Institute for Health Research Biomedical Research Centres funding scheme.

## Supplementary Material

## References

[1] Antinori A Lazzarin A Uglietti A Palma M Mancusi D Termini R. Efficacy and safety of boosted darunavir-based antiretroviral therapy in HIV-1-positive patients: results from a meta-analysis of clinical trials. *Sci Rep* 2018; 8:5288. 29588457 10.1038/s41598-018-23375-6 PMC5869729

[2] World Health Organization. Consolidated guidelines on HIV prevention, testing, treatment, service delivery and monitoring: recommendations for a public health approach. Geneva; 2021. 34370423

[3] Orkin C DeJesus E Khanlou H Stoehr A Supparatpinyo K Lathouwers E. Final 192-week efficacy and safety of once-daily darunavir/ritonavir compared with lopinavir/ritonavir in HIV-1-infected treatment-naïve patients in the ARTEMIS trial. *HIV Med* 2013; 14:49–59. 10.1111/j.1468-1293.2012.01060.x 23088336

[4] Martinez E Gonzalez-Cordon A Ferrer E Domingo P Negredo E Gutierrez F. Differential body composition effects of protease inhibitors recommended for initial treatment of HIV infection: a randomized clinical trial. *Clin Infect Dis* 2015; 60:811–820. 25389256 10.1093/cid/ciu898

[5] Lennox JL Landovitz RJ Ribaudo HJ Ofotokun I Na LH Godfrey C. Efficacy and tolerability of 3 nonnucleoside reverse transcriptase inhibitor-sparing antiretroviral regimens for treatment-naive volunteers infected with HIV-1: a randomized, controlled equivalence trial. *Ann Intern Med* 2014; 161:461–471. 25285539 10.7326/M14-1084 PMC4412467

[6] HIV New Product Introduction Toolkit: CHAI DRV/r 2L Product Profile. 2022. Available at: https://www.newhivdrugs.org/post/chai-drv-r-product-profile.

[7] World Health Organization. Overview of WHO recommendations on HIV and sexually transmitted infection testing, prevention, treatment, care and service delivery. Geneva; 2025.

[8] World Health Organization. Optimization of second-line and third-line antiretroviral therapy for people living with HIV: meeting report, 27–28 November 2023. Geneva; 2024.

[9] Williams PL Yildirim C Chadwick EG Van Dyke RB Smith R Correia KF. Association of maternal antiretroviral use with microcephaly in children who are HIV-exposed but uninfected (SMARTT): a prospective cohort study. *Lancet HIV* 2020; 7:e49–e58. 31740351 10.1016/S2352-3018(19)30340-6 PMC6952580

[10] Smith C Silveira L Crotteau M Garth K Canniff J Fetters KB. Modern antiretroviral regimens in pregnant women: virologic outcomes and durability. *AIDS* 2024; 38:21–29. 37289582 10.1097/QAD.0000000000003616 PMC11375792

[11] Patel K Huo Y Jao J Powis KM Williams PL Kacanek D. Dolutegravir in pregnancy as compared with current HIV regimens in the United States. *N Engl J Med* 2022; 387:799–809. 36053505 10.1056/NEJMoa2200600 PMC9744124

[12] Gacic N Tulloch K Money D Tkachuk S. Daily ritonavir-boosted darunavir for viral suppression in pregnancy (DRV-P). *HIV Med* 2024; 25:129–134. 37816686 10.1111/hiv.13546

[13] Gilleece DY Tariq DS Bamford DA Bhagani DS Byrne DL Clarke DE. British HIV Association guidelines for the management of HIV in pregnancy and postpartum 2018. *HIV Med* 2019; 20 (Suppl 3):s2–s85. 10.1111/hiv.12720 30869192

[14] Panel on Treatment of Pregnant Women with HIV Infection and Prevention of Perinatal Transmission. Recommendations for use of antiretroviral drugs in pregnant HIV-1-infected women for maternal health and interventions to reduce perinatal HIV transmission in the United States. 2025.

[15] Ambrosioni J Levi L Alagaratnam J Van Bremen K Mastrangelo A Waalewijn H. Major revision version 12. 0 of the European AIDS Clinical Society guidelines 2023. *HIV Med* 2023; 24:1126–1136. 37849432 10.1111/hiv.13542

[16] Stek A Best BM Wang J Capparelli EV Burchett SK Kreitchmann R. Pharmacokinetics of once versus twice daily darunavir in pregnant HIV-infected women. *J Acquir Immune Defic Syndr* 2015; 70:33–41. 25950206 10.1097/QAI.0000000000000668 PMC4579345

[17] Crauwels HM Kakuda TN Ryan B Zorrilla C Osiyemi OO Yasin S. Pharmacokinetics of once-daily darunavir/ritonavir in HIV-1-infected pregnant women. *HIV Med* 2016; 17:643–652. 27187894 10.1111/hiv.12366

[18] Schalkwijk S Ter Heine R Colbers A Capparelli E Best BM Cressey TR. Evaluating darunavir/ritonavir dosing regimens for HIV-positive pregnant women using semi-mechanistic pharmacokinetic modelling. *J Antimicrob Chemother* 2019; 74:1348–1356. 30715324 10.1093/jac/dky567 PMC6477987

[19] Murtagh R Else LJ Kuan KB Khoo SH Jackson V Patel A. Therapeutic drug monitoring of darunavir/ritonavir in pregnancy. *Antivir Ther* 2019; 24:229–233. 30728322 10.3851/IMP3291

[20] Eke AC Stek AM Wang J Kreitchmann R Shapiro DE Smith E. Darunavir pharmacokinetics with an increased dose during pregnancy. *J Acquir Immune Defic Syndr* 2020; 83:373–380. 31923087 10.1097/QAI.0000000000002261 PMC7258985

[21] Curran A, Ocana I, Deig E. Darunavir/ritonavir once daily total and unbound plasmatic concentrations in HIV-infected pregnant women. Fourteenth International Workshop on Clinical Pharmacology of HIV Therapy; Amsterdam, Netherlands: Reviews in Antiviral Therapy and Infectious Diseases; 2013.

[22] Crauwels HM Osiyemi O Zorrilla C Bicer C Brown K. Reduced exposure to darunavir and cobicistat in HIV-1-infected pregnant women receiving a darunavir/cobicistat-based regimen. *HIV Med* 2019; 20:337–343. 30873741 10.1111/hiv.12721

[23] Momper JD Wang J Stek A Shapiro DE Scott GB Paul ME. Pharmacokinetics of darunavir and cobicistat in pregnant and postpartum women with HIV. *AIDS* 2021; 35:1191–1199. 34076612 10.1097/QAD.0000000000002857 PMC8173003

[24] Paioni P Capaul M Brunner A Traytel A Aebi-Popp K Crisinel PA. Cohort profile: the Swiss Mother and Child HIV Cohort Study (MoCHiV). *BMJ Open* 2024; 14:e086543. 10.1136/bmjopen-2024-086543 PMC11418562 39313283

[25] Colbers A Moltó J Ivanovic J Kabeya K Hawkins D Gingelmaier A. Pharmacokinetics of total and unbound darunavir in HIV-1-infected pregnant women. *J Antimicrob Chemother* 2015; 70:534–542. 25326090 10.1093/jac/dku400

[26] Taylor GP Clayden P Dhar J Gandhi K Gilleece Y Harding K. British HIV Association guidelines for the management of HIV infection in pregnant women 2012. *HIV Med* 2012; 13 (Suppl 2):87–157. 22830373 10.1111/j.1468-1293.2012.01030_2.x

