# Supplementary material for: Dosing of ritonavir-boosted darunavir for treatment of HIV in pregnancy
Source: AIDS. 2026 Feb 11;40(6):831–9. doi: 10.1097/QAD.0000000000004461 (PMC13152046; doi:10.1097/QAD.0000000000004461)
Supplement: Supplementary file 1 [file aids-40-831-s001.docx]

**Supplementary Table.** Pregnancy characteristics by delivery viral load, *N*=189

|  | **Delivery VL <50 copies/mL,**  *N*=175 | **Delivery VL ≥50 copies/mL,**  *N*=14 | *p*-value |
| --- | --- | --- | --- |
| **Age at delivery, years, *n* (%)** |  |  |  |
| <25 years | 4 (2.3) | 0 (0.0) |  |
| 25-34 years | 86 (49.1) | 10 (71.4) |  |
| ≥35 years | 85 (48.6) | 4 (28.6) | 0.336 |
| **Cohort country, *n* (%)** |  |  |  |
| Romania | 2 (1.1) | 1 (7.1) |  |
| Switzerland | 49 (28.0) | 1 (7.1) |  |
| United Kingdom | 124 (70.9) | 12 (85.7) | 0.070 |
| **Timing of HIV diagnosis, *n* (%)** |  |  |  |
| Before this pregnancy | 157 (89.7) | 12 (85.7) |  |
| During this pregnancy | 18 (10.3) | 2 (14.3) | 0.647 |
| **ART timing, *n* (%)** |  |  |  |
| Conceived on ART | 142 (81.1) | 8 (57.1) |  |
| Started ART in 1st/2nd trimester | 27 (15.4) | 5 (35.7) |  |
| Started ART in 3rd trimester | 6 (3.4) | 1 (7.1) | 0.057 |
| **DRV group, *n* (%)** |  |  |  |
| DRV at conception | 121 (69.1) | 6 (42.9) |  |
| Other ART at conception, switched to DRV/r | 21 (12.0) | 2 (14.3) |  |
| Started ART in pregnancy (DRV/r) | 30 (17.1) | 6 (42.9) |  |
| Started ART in pregnancy (other ART, switched to DRV/r) | 3 (1.7) | 0 (0.0) | 0.093 |
| **DRV/r dose, *n* (%)** |  |  |  |
| 800/100 mg once daily | 154 (88.0) | 8 (57.1) |  |
| 600/100 mg twice daily | 21 (12.0) | 6 (42.9) | 0.007 |
| **First CD4 count in pregnancy, cells/mm^3^, median (IQR)** (*n*=184) | 522 (382-670) | 372 (165-499) | 0.020 |
| **First CD4 count in pregnancy, *n* (%)** (*n*=184) |  |  |  |
| <350 cells/mm^3^ | 38 (22.4) | 6 (42.9) |  |
| ≥350 cells/mm^3^ | 132 (77.6) | 8 (57.1) | 0.104 |

ART, antiretroviral therapy; DRV, darunavir; DRV/r, ritonavir-boosted darunavir; VL, viral load
